# Painful penile erection as a manifestation of metastatic pancreatic ductal adenocarcinoma

**DOI:** 10.1007/s12328-025-02278-w

**Published:** 2026-01-14

**Authors:** Haruki Mori, Hiromitsu Maehira, Nobuhito Nitta, Masanori Shiohara, Takeru Maekawa, Toru Miyake, Sachiko Kaida, Masatsugu Kojima, Katsushi Takebayashi, Masaji Tani

**Affiliations:** 1 Department of Surgery, Shiga University of Medical Science, Setatsukinowa-chou, Otsu, Shiga 520-2192 Japan; 2 Department of Pathology, Shiga University of Medical Science, Otsu, Shiga Japan

**Keywords:** PDAC, Penile metastasis, KRT7 (CK7), KRT20 (CK20)

## Abstract

**Background:**

Penile metastasis represents one of the rarest metastatic sites despite the organ’s rich vascular network. Most reported cases originate from malignancies of the genitourinary tract, whereas metastasis from pancreatic ductal adenocarcinoma (PDAC) is exceedingly uncommon. Primary tumors responsible for penile metastases are typically located in the prostate or rectum and are frequently associated with disseminated disease and poor prognosis.

**Case presentation:**

An 86-year-old man presenting with abdominal pain underwent distal pancreatectomy with regional lymphadenectomy for PDAC. Three months after surgery, computed tomography revealed multiple liver metastases, para-aortic lymph node involvement, and peritoneal dissemination. Subsequently, the patient developed penile pain and persistent erection. Physical examination revealed multiple whitish indurated nodules on the glans penis. Ultrasonography demonstrated a hypoechoic mass, and a penile biopsy confirmed metastatic adenocarcinoma consistent with the primary pancreatic lesion based on morphological features and KRT7/20 immunoprofiles. The patient’s condition progressively deteriorated, and he died five months after surgery.

**Conclusion:**

Penile metastasis from PDAC is an extremely rare event and portends a dismal prognosis. Including our patient, only six cases have been documented to date. Awareness of this manifestation is important, as penile symptoms such as pain or persistent erection in patients with PDAC may indicate advanced systemic dissemination.

## Introduction

Pancreatic ductal adenocarcinoma (PDAC) is characterized by an aggressive biological behavior and a high recurrence rate even after curative resection. Although surgical resection remains the only potentially curative treatment, recurrence commonly occurs in the liver, peritoneum, lungs, or at the surgical margin [1–3]. Metastatic spread of PDAC to the penis is exceptionally rare despite the organ’s rich vascularization and extensive lymphovascular connections with adjacent pelvic structures [4]. To date, approximately 500 cases of penile metastatic disease have been reported in the literature, the majority originating from malignancies of the genitourinary tract, followed by rectal, gastrointestinal, and pulmonary cancers [5, 6]. Penile metastasis typically manifests in the terminal phase of disseminated malignancy, with a median survival of less than one year after diagnosis [4]. The clinical manifestations of penile metastasis vary and may include palpable masses (55%), persistent erection (31%), and dysuria (21%) [7]. Metastatic involvement usually affects the corpus cavernosum more frequently than the corpus spongiosum; however, reports of penile metastasis from pancreatic cancer are extremely limited, and its clinicopathological features and metastatic routes remain poorly understood.

We herein describe a rare case of penile metastasis arising from PDAC and discuss its clinical presentation and diagnostic implications in the context of previously reported cases.

## Case presentation

An 86-year-old man was admitted to Shiga University of Medical Science Hospital with complaints of abdominal pain. Laboratory tests revealed a normal complete blood count and normal liver function. Serum tumor markers showed a carcinoembryonic antigen (CEA) level of 9.3 ng/mL (normal range, < 5 ng/mL), a carbohydrate antigen 19− 9 (CA19-9) level of 1084 U/mL (normal range, < 37 U/mL), and a Duke pancreatic monoclonal antigen type 2 (DUPAN-2) level of 140 U/mL (normal range, < 150 U/mL). Computed tomography (CT) demonstrated a low-density mass in the pancreatic body, and the patient was diagnosed with pancreatic ductal adenocarcinoma (PDAC).

Distal pancreatectomy with regional lymphadenectomy was performed. Cytology of the intraoperative peritoneal lavage showed no malignant cells. The postoperative course was complicated by a grade B pancreatic fistula, as defined by the International Study Group on Pancreatic Fistula (ISGPF) criteria, which was successfully managed with percutaneous drainage. The patient was discharged on postoperative day 47. Histopathological examination revealed a moderately differentiated ductal adenocarcinoma in the pancreatic body with retroperitoneal and neural plexus invasion, with invasion into the retropancreatic tissue abutting the posterior pancreatic surface. No lymphatic or venous invasion was identified on immunohistochemical/histochemical staining (ly0 by D2-40; v0 by Elastica–van Gieson [EVG]); however, six regional lymph nodes were positive for metastasis. The final pathological stage was pT3N1M0 (Stage IIB). Postoperative adjuvant chemotherapy was not administered due to concerns regarding postoperative quality of life.

**Fig. 1:**
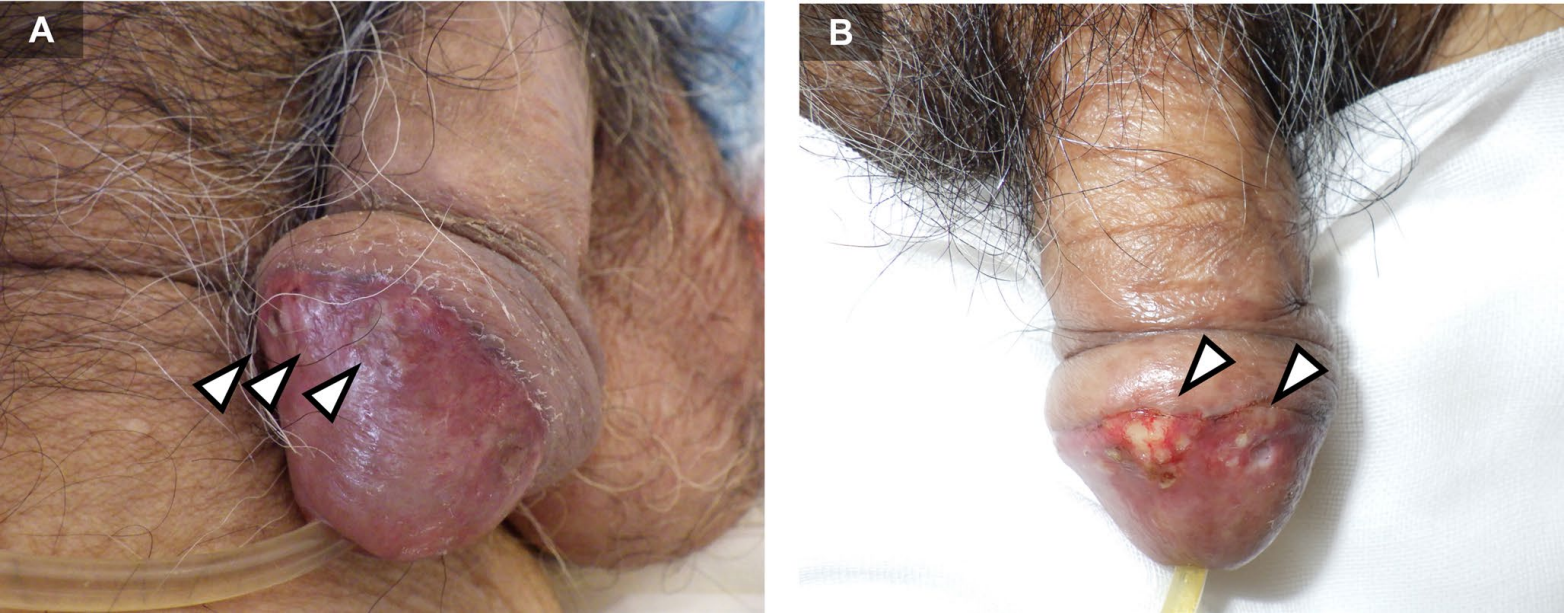
Appearance of penis photographs (**A**,** B**). The penis was hard and erect, and the glans showed a whitish indurated lesion (arrowhead)

**Fig. 2:**
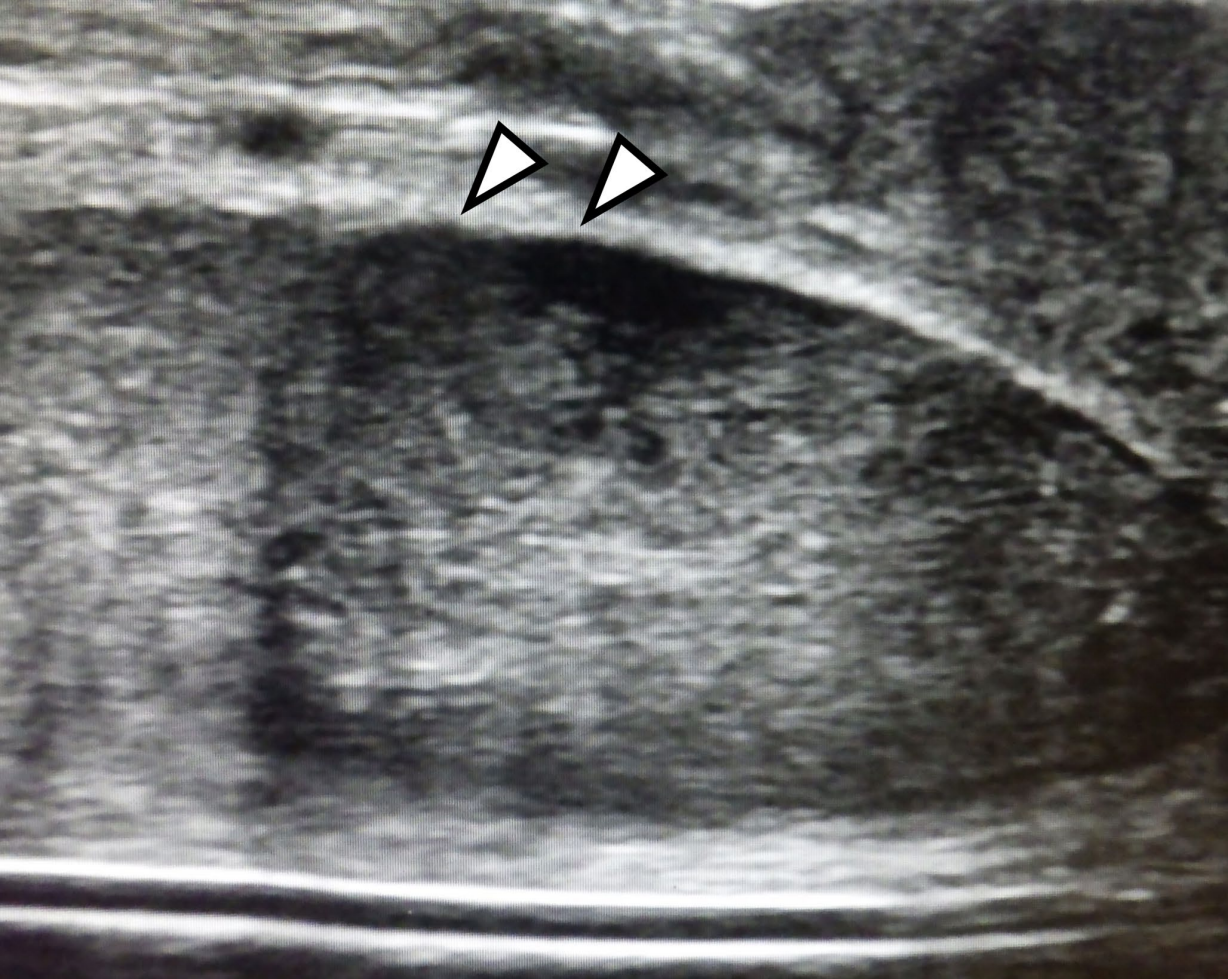
Ultrasonography revealed the white tone indurated lesion at the glans as a hypoechoic area (arrowhead)

**Fig. 3:**
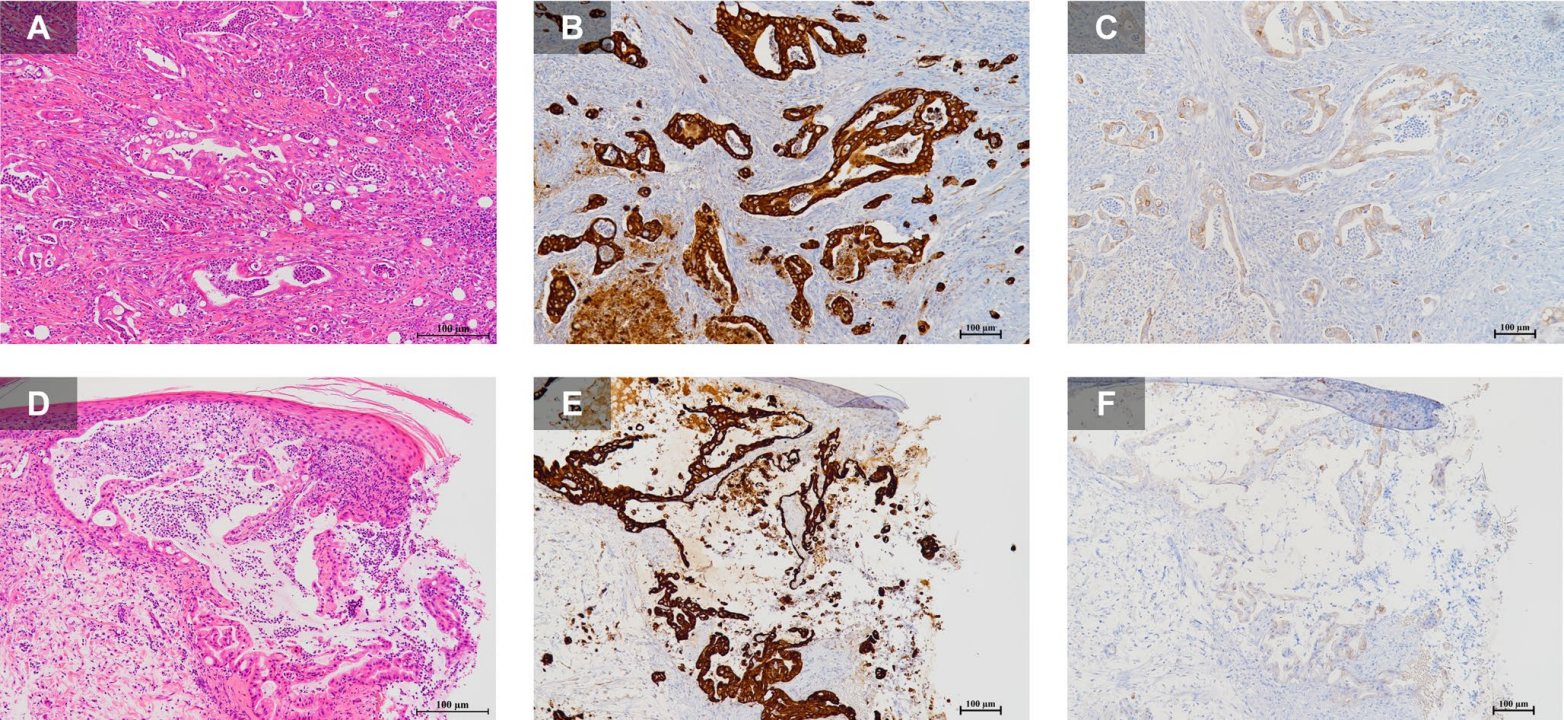
H&E stained images (**A**,** D**) and immunohistochemical stained images of KRT7 (**B**,** E**) and KRT20 (**C**,** F**). The histology of the primary lesion (**A**) and penile metastasis (**D**) was very similar. Immunohistochemistry was consistent with KRT7-positive (**B**,** E**) and KRT20-partially positive (**C**,** F**)

Approximately three months after surgery, CT revealed multiple pulmonary nodules, liver metastases, para-aortic lymph node metastases, and peritoneal dissemination. The patient declined systemic chemotherapy. Four months postoperatively, he developed respiratory distress, dysuria, severe penile pain, and persistent penile erection. Physical examination revealed multiple whitish, indurated nodules on the glans penis (Fig. 1A, B). Ultrasonography demonstrated a hypoechoic mass (Fig. 2). Penile biopsy revealed adenocarcinoma cells with enlarged, pleomorphic nuclei and glandular tubular structures within the dermis, morphologically consistent with the primary pancreatic lesion (Fig. 3A, D). Immunohistochemical staining showed identical KRT7 (CK7) and KRT20 (CK20) expression patterns between the primary and metastatic lesions (Fig. 3B, C, E, F).

Symptomatic relief was achieved with urethral balloon placement. The patient’s general condition gradually deteriorated, and he died five months after surgery.

## Discussion

Penile metastasis was first described by Eberth in 1870 [8]. Despite the penis being a highly vascularized organ with extensive lymphovascular connections to adjacent pelvic structures, metastatic involvement of the penis remains exceedingly rare [4, 9]. The most common primary sites are urogenital organs such as the bladder, prostate, and kidney, which together account for approximately 70% of cases, followed by colorectal cancers (21%) [10].

Penile metastasis typically presents with penile induration or nodules, persistent erection, penile pain, and lower urinary tract symptoms such as dysuria or urinary retention [6, 9, 10]; thrombosis of the dorsal penile vein has also been reported as an initial clue [4]. Clinicians should suspect metastatic involvement when new-onset penile pain, persistent erection, or a palpable induration develops in a patient with a known advanced malignancy [6, 9, 10]. In pancreatic ductal adenocarcinoma, published cases describe perineal discomfort, erection-associated penile pain, firm nodules, and diffuse induration of the coronal sulcus as presenting features, with palliative measures such as catheterization and analgesia or radiotherapy typically selected according to performance status and disease extent [4]. In our patient, the constellation of severe penile pain, persistent erection, dysuria, and whitish indurated nodules of the glans prompted biopsy and confirmed metastatic pancreatic ductal adenocarcinoma, underscoring that attentive assessment of penile symptoms can expedite diagnosis and facilitate symptom-directed care.

Painful erection and malignant priapism associated with penile metastasis are most commonly attributable to veno-occlusive dysfunction caused by neoplastic infiltration of the corpora cavernosa and involvement of the dorsal venous system, which impairs venous egress and precipitates cavernosal ischemia [9, 11, 12]. Although urethral catheterization does not reverse priapism, it alleviates bladder outlet obstruction and perineal/urethral discomfort. Durable detumescence typically requires cavernosal aspiration followed by intralesional sympathomimetic injection. If these measures are insufficient, shunt procedures are indicated. In advanced disease, palliative radiotherapy or penectomy may be appropriate, with selection individualized to performance status and the extent of systemic involvement [13, 14].

Computed tomography (CT) and magnetic resonance imaging (MRI) are useful modalities for detecting metastatic penile tumors. On contrast-enhanced CT, metastatic lesions typically exhibit strong enhancement and are well demarcated from surrounding tissues [15]. MRI findings usually demonstrate low-intensity lesions on both T1- and T2-weighted images with nonspecific gadolinium enhancement [16]. In the present case, given the patient’s poor general condition, CT and MRI could not be performed, and ultrasonography alone was used to evaluate the lesion (Fig. 2). Additional immunohistochemical markers (e.g., CDX2, MUC1, MUC2, TTF-1, PSA) were not assessed. The metastatic lesion closely mirrored the pancreatic primary in gland-forming architecture, nuclear atypia, and desmoplastic stroma; together with the available immunophenotype (KRT7/KRT20) and the clinical context, these features support a pancreatic origin (Fig. 3), a pattern typically observed in PDAC [17, 18]. The patient had no prior history of other malignancies, confirming the diagnosis of penile metastasis from PDAC.

Penile metastasis generally occurs in the terminal stage of systemic disease, and the prognosis is extremely poor, with most patients dying within one year of diagnosis [4, 5, 19]. Therapeutic strategies are mainly palliative. Surgical interventions, including partial or total penectomy, rarely improve survival outcomes [4]. In our case, palliative management with urethral catheter placement provided symptomatic relief of penile pain and dysuria; however, the patient died one month after the diagnosis of penile metastasis.

A targeted literature review using PubMed and supplementary sources identified five previously reported cases of penile metastasis from pancreatic ductal adenocarcinoma. Including our patient, at least six cases have been documented to date, and these are summarized in Table 1 [4, 20–23]. The mechanism of penile metastasis from PDAC remains unclear. Proposed routes include hematogenous or lymphatic dissemination, direct extension from peritoneal seeding, and secondary invasion through adjacent organs such as the colon [24, 25]. Interestingly, in all reported cases including ours, the primary pancreatic tumor was located in the body or tail of the pancreas. Although the precise pathway of spread to the penis remains uncertain, retrograde venous and retrograde lymphatic routes have been implicated [9]. In our patient, retroperitoneal invasion and regional lymph-node metastases suggest that venous return via the retroperitoneal and pelvic plexuses and/or lymphatic connections to the internal iliac chains may have contributed to penile seeding [26]. Further case accrual is warranted to elucidate these mechanisms.

**Table 1:** Reported cases of penile metastasis from pancreatic ductal carcinoma

	Author	Year	Age/sex	PDAC (location)	Surgery	Metastatic lesion	Presenting symptoms	Management	Survival time (month)
1	Hashimoto [20]	1989	50/M	Pt	None	Liver Prostate	Penile pain	Chemotherapy	2 m death
2	Ahn [21]	1997	60/M	Pt	None	Peritoneal dissemination	Penile pain Malignant priapism	None	4 m death
3	Tello [22]	2012	67/M	Pb	None	Bone Pelvic lymph node	Penile pain Malignant priapism Dysuria	Palliative radiation	3 m alive
4	Virdis [4]	2019	66/M	Pt	DP	Bone Liver	Penile pain Malignant priapism	Chemotherapy	9 m death
5	Yakut [23]	2022	49/M	Pt	None	Liver	Penile mass	Chemotherapy Penile mass excision	1 m alive
6	Our case	2025	86/M	Pb	DP	Lung Peritoneal dissemination	Penile pain Malignant priapism Dysuria	Urethral catheter Analgesia	5 m death

F, Female: M, Male: Pb, Pancreatic body: PDAC, Pancreatic ductal adenocarcinoma: Pt, Pancreatic tail

## Conclusion

Although penile metastasis from pancreatic cancer is rare, it represents a manifestation of advanced systemic disease and a marker of terminal progression. Despite its poor prognosis, this condition can cause severe distress due to symptoms such as persistent erection, penile pain, and dysuria, all of which markedly impair the patient’s quality of life. Palliative measures—such as analgesia and urethral catheter placement—may therefore be essential to alleviate symptoms and maintain comfort during end-stage disease.
